# Construction Materials from Vitrified Lignite Fly Ash in Plasmatron Plasma Reactor

**DOI:** 10.3390/ma12060905

**Published:** 2019-03-19

**Authors:** Jakub Szałatkiewicz

**Affiliations:** Przemysłowy Instytut Automatyki i Pomiarów PIAP, al. Jerozolimskie 202, Warszawa 02-486, Poland; jszalatkiewicz@piap.pl or jakub.szalatkiewicz@gmail.com

**Keywords:** plasma, vitrification, waste, fly ash, lignite, synthetic construction aggregate

## Abstract

This article presents results of an investigation of vitrified (melted) fly ash samples from lignite (brown coal) in a plasmatron plasma reactor, to determine its mechanical and chemical properties. The XRF elemental analysis results of sample tests, from before the vitrification process and after the vitrification process are shown. The experiments were carried out in a plasma plasmatron reactor with a total power of 65 kW, enabling testing on a quarter technical scale. During the tests, samples of fly ash of about 4 kg mass were processed under selected process conditions. Produced samples of vitrified materials were analyzed in accordance to the requirements for building/construction materials. Results from this investigation confirm its quality to be used as concrete and cement filler, as an addition, and as synthetic aggregate, safe for the environment and neutral for cements. Also the most important leaching of heavy metals to water was analyzed which confirmed meeting of all of requirements necessary to use this material in building materials.

## 1. Introduction

Fly ash, and especially lignite/brown coal fly ash, as it is called in Poland—is becoming the main waste from thermal power plants, as in Europe where hard coal use is being reduced and lignite-fired power plants still operate. Lignite fly ash, due to its significant amounts, is a major problem as it is in most cases landfilled in ash ponds, which in some circumstances causes environmental hazard [1]. Many authors have investigated various options of recycling and potential applications of this waste, including as a component in cement, mortar, concrete, bricks, and tile forming [2]. It could also be used as a resource, for the recovery of valuable elements i.e., rare earth elements [3], and even as a fertilizer addition to improve soil quality and productivity [4]. Other applications of brown coal fly ash as sorbents for the removal of synthetic dyes from waters are present in the literature [5]. All of the above applications are potential threats to the environment and human health, as fly ash may release toxic substances and heavy metals when in contact with water. 

In the literature various paths of examination are exercised, due to the power sector’s need for the utilization of huge amounts of lignite fly ash, as landfilling becomes difficult and generates additional costs. That is why the author of this article carried out an examination of alternative approaches to fly ash utilization, namely the application of plasma technology to vitrify two exemplary samples of lignite fly ash from Polish lignite/brown coal fired power plants. 

Vitrification of ashes is one of the methods allowing for its utilization, potential use, management, and potentially the recovery of resources. It involves bringing the fly ash to the point of melting by heating it to a high temperature exceeding its melting point. There are known solutions for fly ash and bottom ash vitrification using the chemical energy provided by burning fuel in oxygen [6,7] and through the various use of plasma [8]. In contrast to vitrification conducted using oxygen combustion, plasma vitrification involves usage of plasma as the main source of heat in the process. Plasma vitrification of ashes may be accomplished through the use of plasma directly inside the reactor produced by an electric arc between the electrodes [8] i.e., DC arc furnace, and through the use of plasmatrons, which are plasma generators, in which the plasma stream is produced and delivered to the reactor. The main product of the vitrification process is “stone like” material, flowing out from the reactor at a temperature of about 1400–1600 °C—similar to volcano lava. 

**Figure 1:**
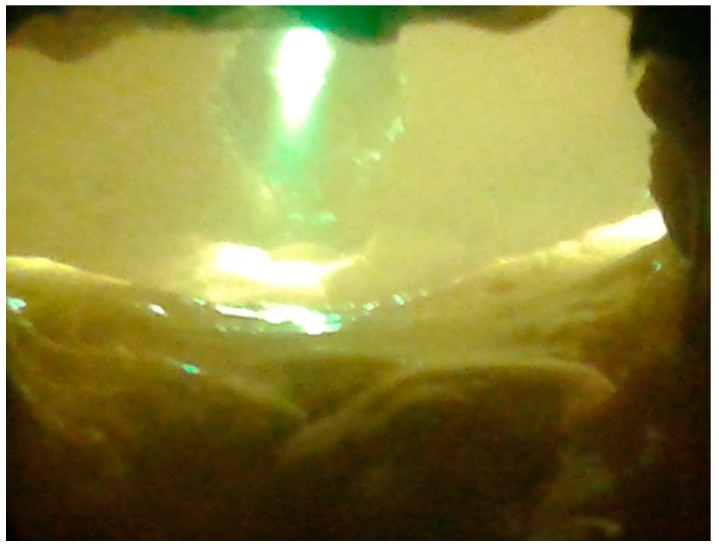
Plasma reactor chamber during operation. In the middle is a visible plasma torch during operation. The picture taken through a light filter, with a temperature in the reactor about 1450 °C.

**Figure 2:**
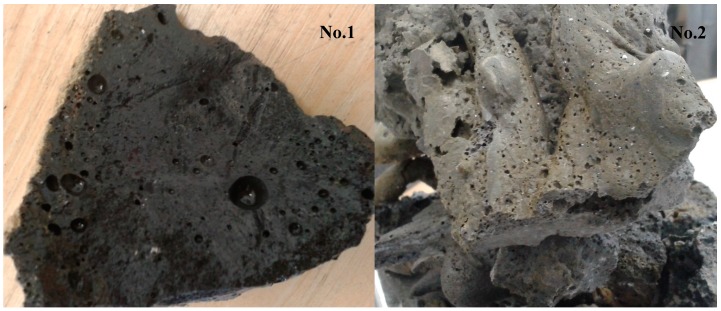
Examples of cooled down vitrified lignite fly ash samples no.1 and 2.

An example of vitrified molten material flowing out from the reactor, and the interior of the plasma reactor, is presented on Figure 1. The vitrified fly ash product, depending on the process conditions, cooling method, and ash composition has certain properties, but in end is a stable, safe, and similar to glass or rock. Examples of the appearance of vitrified fly ash are presented in Figure 2. This photograph shows the molten fly ash obtained from the presented studies. This particular form of vitrified fly ash presented in the current study is dense, hard, and with a minimal number of gas bubbles. However, by applying different process conditions the vitrified fly ash can be obtained in the form of foam, where gas bubbles are dominating the material structure, and are trapped in the vitrified fly ash mass. 

## 2. Materials and Methods

**Figure 3:**
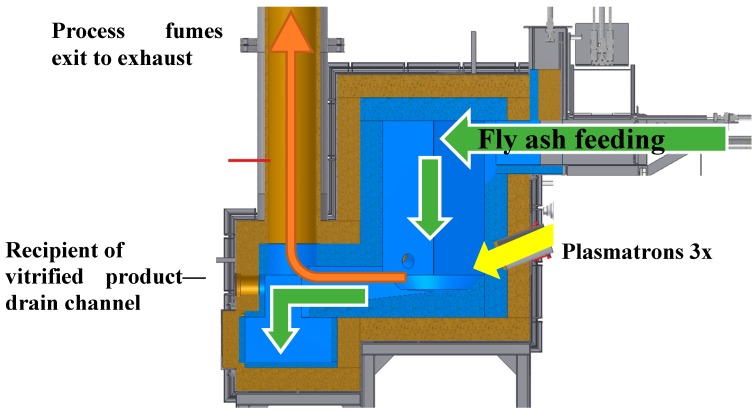
Cut through plasma reactor. Arrows indicate the material, fumes, and plasma flow.

Presented research was carried out in a plasmatron plasma reactor located in the Industrial Institute of Automation and Measurements in Warsaw, Poland, in which three plasma sources in the form of plasmatrons were placed at its circumference at 120°. Plasmatrons nozzle axis outlet was located in the lower part of the reactor chamber. The reactor cross-section is shown in Figure 3.

The only source of heat in this reactor are three 20 kW arc plasmatrons. Plasmatrons achieve up to 80% thermal efficiency of the conversion of electric energy into plasma, and in relation to the energy consumed from the grid, after taking into account the efficiency of the power supply, their total efficiency is about 70%. Plasmatrons produce plasma jets from electricity and working gas, which in this case was technical compressed air.

The construction of the reactor allows the maintenance of temperatures up to 1650 °C. The temperature inside the chamber is measured at a distance of 30 mm from the wall, at a height of 350 mm from the bottom of the reactor. In the zone of direct effect of the plasma burners on the material in the chamber, the temperature is many times higher than the temperature measured in the reactor volume. This is because each of the three plasmatrons generate a plasma stream with a temperature of over 11,000 °C [9].

### 2.1. Lignite Fly Ash Samples

**Figure 4:**
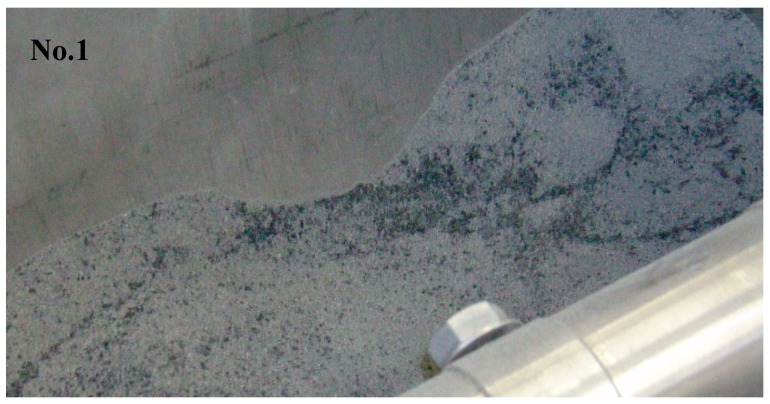

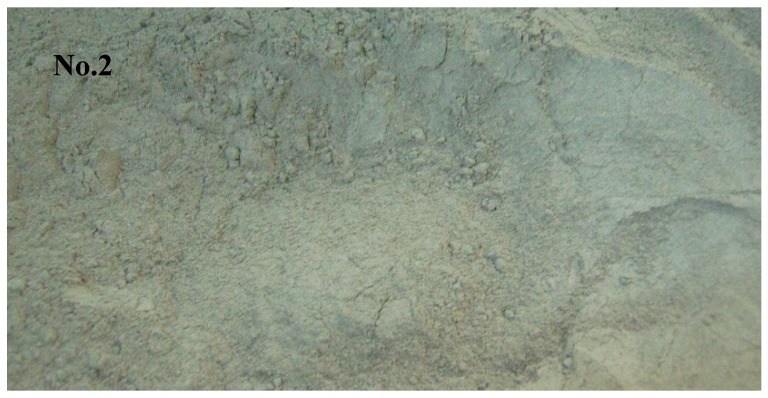
Photograph of lignite fly ash samples, no. 1 and 2 before the process.

The subject of the research were samples of fly ash from the combustion of lignite (brown coals) from Polish power plants. The samples were dry (moisture of about 0.2%), and uniform in the form of small grain size particles. Exemplary samples photographs are presented on Figure 4.

The samples were analyzed by a standardless XRF method allowing identification and quantification of key elements in them. The results are presented in Table 1. 

The samples differ mainly in the content of silica, which amount in ash no. 1, is 10% more than in the ash sample no. 2. Also a significant difference between samples is in the content of aluminum oxide and iron oxide, which in the sample no. 2, is almost twice as much than in sample no. 1. Next the calcium content in the tested samples is similar for both samples. 

Samples also differ by more than ten times in Na_2_O and K_2_O. However, their share in the total mass of ashes is of the order of 1%, therefore this difference has very limited impact on the conducted research and results. 

Similarly, in the case of sulfur designated as SO_3_ in sample no 1, its quantity is higher than in sample no. 2, and amounts to 2.9% in sample no. 2, and 1.21% in sample no 1. 

In summary, the tested samples differ the highest degree of content of silica and aluminum oxide. In sample no. 1 the SiO_2_ amount is 22.3%, and in the sample no. 2 it is 36.5%, while Al_2_O_3_ occurs in a smaller amount in sample no. 1, 10.4%, than in the sample no. 2, which contain 17.4%. The remaining compounds in the tested samples are at a similar level, varying within 1–2%: this difference has no significant effect on the obtained results and process parameters.

### 2.2. Vitrification Process Description

The ash was introduced to the preheated reactor at an ambient temperature, and its initial temperature was about 20 °C. Throughout the process, exhaust gases leaving the reactor carrying out heat from it, which is lost from the process. The temperature of the exhaust gases leaving the reactor is over 1200 °C.

The vitrification tests were carried out according to the following scheme. The test stand was started and the reactor was heated up to the operating temperature of about 1410 °C, after it was reached, ash feeding was started with the screw conveyor.

The end of ash feeding followed the filling of the form/mold in which the vitrified material was collected. Then, the vitrified material remained in the reactor chamber was pulled out mechanically with the steel rod. After which the power of the plasmatrons was switched off and the whole test stand was cooled. The reactor and vitrified material was left to cool down. Then it was removed from the reactor and from the mold, and its mass was determined and samples taken for further analysis.

The full test procedure takes an average of 48 h for each experiment.

## 3. Results and Discussion

### 3.1. Experimental Results

Both samples no. 1 and 2, were vitrified in air atmosphere, as plasma was generated using compressed air. Usage of air was about 14 Nm^3^/h.

**Figure 5:**
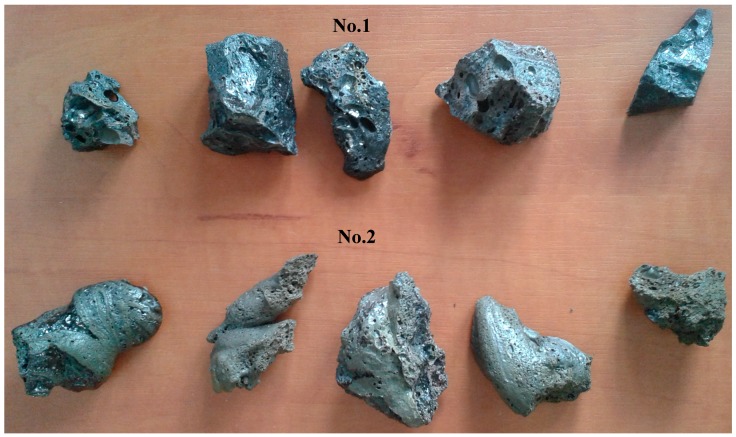
Picture presenting crushed vitrified lignite fly ash samples no. 1 and no.2.

Figure 2 and Figure 5 presents pictures of obtained vitrified samples after the process. 

Below in the Table 2, are presented process parameters and conditions for each sample. 

Both vitrified samples are fully melted, homogeneous, in the form of hard, solid-rock glass, however, some areas are partially spongy with sealed gas bubbles. There is no dusting, no ash is visible, and they shape to the mold in which they have settled. It is possible to crumble them in order to fragment them, although the hardness of the vitrified samples is higher than >7 (quartz) in Mohs scale of hardness [10] which means the hardness of the vitrified fly ash is very high.

Color of the sample no. 1 is black and the sample no. 2 is rather gray/green inside, and on top slightly brown. 

The sample no. 2 is very hard to crush; even a hammer does not crush the rock and it requires usage of significant force to crush it to pieces. Sample no. 1 is easier to crush, and it has a form closer to glass.

### 3.2. Elemental Analysis of Vitrified Samples

Collected samples after vitrification were analyzed by XRF standardless analysis to quantify the main elemental composition after the process. The results are summarized in Table 3. Presented results allow analysis of the elemental composition change as an effect of the carried out plasma vitrification process. 

As presented in Table 1 and Table 3, the elemental composition of the analyzed samples, as a result of the vitrification process, has not changed significantly. Most of the compounds the elements contained in the ash are present in very similar amounts in both vitrified and raw samples. The largest difference is in the case of SiO_2_ of the ash from the sample no. 1, and for Al_2_O_3_ of the ash of sample No. 2. Sample no. 1, before the process, contained SiO_2_ in the amount of 36.6%, and in the vitrified sample it was 32.5%. For sample no. 2, there was a difference in the content of Al_2_O_3_: in the raw fly ash it was 17.4%, and in the vitrified sample it was 13.7%. The origin of this change can be explained by a sampling error as fly ash sample investigated before the process (jar of ash powder) is not the same as the fragmented rock after the process which was milled before the XRF analysis. 

It is interesting to notice decrement of the sulfur content in the vitrified material, which for both samples is about 0.1% regardless of its level in the raw sample. This means that the sulfur in feedstock (fly ash) was released to the flue gas and removed from the sample during the process. This mechanism is also responsible for mass loss of the processed material during the vitrification process in the plasmatron plasma reactor.

The collected data indicate that in both experiments, the mass of vitrified material is smaller than the mass of the ash introduced into the reactor. On one hand, this may be due to the release of volatile substances from the ashes (combustion of the carbon, release of potentially bonded CO_2_, release of sulfur, moisture, and oxygen release when elements become vitrified). On the other hand, the vitrified product cannot be completely removed from the inside of the reactor after the experiment. A certain amount of processed sample will always stay on the walls of the reactor, on its bottom or in the drain channel. In addition, there are possible losses on the fly-up of ash particles with exhaust fumes, however, observations indicate that fly up is very limited as ash very quickly becomes molten on the surface and forms bigger droplets in the reactor chamber falling down. There is also limited ash dusting during loading, and small loss during molding. Considering the small change in the composition of ash after vitrification, it should be recognized that the estimated reduction in mass due to the ash vitrification process may reach about 5–10% and results are mainly from the removal of flammable and volatile substances.

Examination of the fly ash samples before processing in plasma furnace did not indicate significant amounts of CO_2_ bonded in the fly ash. The examination was based on observation of a raw sample of fly ash when sulfuric acid (H_2_SO_4_ 20%) was dropped over it. No gas release was observed, so no carbon dioxide was released from the fly ash samples. 

Obtained research results indicate that, as a result of the vitrification process, there is no significant change in ash composition. The vitrification process leads to a change in the structure of ashes and the creation of a fairly uniform, solid material.

The compounds contained in the ashes are mainly oxides, which are substances with high chemical, thermal, and inert properties. They can, however, melt, which takes place in the vitrification process, and re-solidify in a new form, additionally binding in their structure other compounds, i.e., heavy metals [11]. Processing of fly ash in plasma reactor at a temperature of over 1450 °C and an oxidizing atmosphere also allows complete combustion and decomposition of, for example, aromatic and other hydrocarbons, which fly ash can contain. 

As part of the work carried out, the density of the obtained vitrified fly ash was also estimated, which turned out to be about 2.7 times higher than the loose ash bulk density before the process of the fly ash. This means that the vitrified material constitutes of only 36% of the volume of the input fly ash. That means that, from 1.2 m^3^ of ash, which mass is a ton, the produced vitrified material will have a volume of about 0.42 m^3^.

### 3.3. Power Consumption

The total energy consumption of the test stand presented in the Table 2, based on the processed ash mass, is a value that exceeds the energy required for the vitrification process, and results from the construction of the test stand. The power consumption of the test stand, regardless of the amount of ash introduced, is about 65 kW. Electricity is consumed to power all of test stand devices and components, including pumps, power supplies, fans, computers, control, and metering. The power consumed from the grid is measured in real time on the power supply of the test stand with a wattmeter. The total energy consumption of the test stand during the test per unit of ash mass is calculated on the basis of the energy consumed at the time when the ash enters the reactor and its mass.

Direct determination of the energy consumption of the vitrification process during the conducted tests turned out to be impossible due to the construction of the stand, whose power is much higher than the energy demand for vitrification. The situation resulted from the fact that during the research the phenomenon delaying the melting of ashes and leading to its accumulation in the reactor in the unmelted form was identified. This phenomenon forced the reduction of the amount of ash being fed to the reactor. The slow heating and ash during the melting process prevented the feeding of a larger amount of cold ash over a longer period of time. An average of 4.9 g/s (423 kg/day) was the highest amount possible to feed, as a higher amount led to ash accumulation in the reactor in an unmelted form. Therefore, with a thermal power of 42 kW, it was possible to vitrify an average of 4.9 g/s of ash, for which theoretically thermal power of 11 kW would be sufficient. The remaining heat supplied to the process was used to raise the reactor temperature, cover the heat losses, and heat up the “air” that was carried out with the exhaust gases. Therefore, the energy consumption of the process was determined indirectly on the basis of the collected data from the conducted tests as well as the parameters and power balances of the test stand.

Taking into account the construction of the test stand, which is not optimized for ash vitrification, but for melting and burning of flammable feedstock, it should be assumed that the average energy consumption of the vitrification process is close to 1 MWh/Mg.

In turn, from calculations of the theoretical amount of heat required to melt them, the heat demand for melting is about 0.6 MWh/Mg of cold ash.

The total energy consumption of the test stand during the tests per mass unit of fly ash was 3.5 and 4.9 MWh/Mg of ash. However, it should be emphasized that the amount of fly ash used in the tests, due to the phenomenon of their long heating and melting, was 2–3 times lower than the available thermal power of the reactor, which results in 2–3 times higher energy absorption obtained in the experiments.

Another important topic in ash vitrification is the occurrence of flammable parts, which leads to the generation of additional heat energy in the vitrification process. However, the amount of energy released as a result of burning a few percent of the contained coal/carbon in ashes, whose mass during the test is about 0.005 kg/s, is so small that it is not possible to measure its impact on the process. However, any addition of fuel in the mass of vitrified ashes leads to a reduction in their mass and volume and to the release of additional energy that partially covers the process’s demand. This phenomenon is also beneficial because instead of electricity to run the process, the primary energy is released in the furnace due to oxidation of fuel, and this energy is supporting in the process and reduce the electricity usage.

## 4. Potential Application of Vitrified Materials Formed from Lignite Fly Ash—Alternative Building Materials and Inert Aggregate for Civil Engineering

Obtained vitrified material was analyzed according to the requirements of PN-EN 12620: 2008—the leaching of heavy metals according to the procedure for building materials. Gathered results were analyzed and compared to the requirements of the PN-EN 12620:2008—aggregates for concrete and building materials which confirmed that permissible limiting values for the leaching of heavy metals and other substances to water are not exceeded. This means that the analyzed material can be used in construction and in building materials in direct contact with water, thus is safe for environment. Results from this investigation are presented in Table 4. 

The next analyzed parameter was alkaline reactivity. The research was carried out according to the test method PN-B-06714-46:1992 Mineral aggregates–testing–determination of the potential alkaline reactivity by the fast method. The test results are presented in Table 5.

The results presented in Table 5 indicate that the artificial material produced from the plasma vitrification process has the lowest alkaline reactivity. Level 0 allows the use of produced vitrified materials as aggregates, and as additions for concretes and cements. 

## 5. Conclusions

Carried out research confirms that plasma vitrification of lignite fly ash is possible in a plasmatron plasma reactor and the results are similar with investigated different samples. 

Detailed elemental analysis reveals that no significant change of the fly ash occurs and produced vitrified fly ash elemental composition is similar to the raw material before the process. 

The power consumption in the process is high due to the construction of test stand, on which the experiments were carried out. However detailed power balance indicate that the overall energy consumption for vitrification of lignite fly ash will be close to 1 MWh/Mg. 

Presented results of carried out investigation of, vitrified fly ash samples, confirm that the vitrified fly ash can be used in construction industry as it:

(1) does not leak any harmful substances to the environment and the material meets all of necessary access criteria for its use as building material in construction industry,
(2) the density of the produced material is above 2 g/cm^3^ which is favorable in construction industry and civil engineering,
(3) the vitrified product is hard, as its hardness is higher than quartz which equals to >7 in Mohs scale,
(4) it is dense: compared to the volume of raw lignite fly ash it is only 36% volume of the input material.

Based on vitrified material properties it can be used as a construction aggregate for roads and in earthworks as a filler for concrete and cements as it is neutral for typical mortars, concretes, and other building materials.

Plasma vitrified fly ash material is hard, does not dust, and is not affected by any environmental conditions. It is also safe for the environment and does not leach any contaminants to the environment. Those benefits allows its broad use in civil engineering applications as artificial/synthetic construction aggregate and as a concrete additive, or filler.

## Figures and Tables

**Table 1:** Elemental analysis of fly ash carried out by XRF standardless analysis, values calculated to oxide form (the results have not been normalized to 100%).

Element	SiO_2_	TiO_2_	Al_2_O_3_	Fe_2_O_3_	MnO	MgO	CaO	Na_2_O	K_2_O	P_2_O_5_	SO_3_
**Sample**	%										
**No. 1**	36.5	1.5	10.4	2.5	0.02	0.6	13.2	0.1	0.1	0.04	0.6
**No. 2**	22.3	2.2	17.4	4.3	0.07	1.1	15.6	1.6	1.2	0.1	2.9

**Table 2:** Process parameters and conditions for each sample.

Vitrification Parameters	Sample No. 1	Sample No. 2
Total average electrical power consumption by the setup during experiment	60 kW	65 kW
Total electrical power consumed by the stand during the experiment, divided by the sample mass	3.5 kWh/kg	4.9 kWh/kg
Fly ash mass in the experiment (feed continuously)	5.5 kg	6 kg
Vitrified mass left after the experiment:	4.3 kg (78% of input mass)	5.2 kg (86% of input mass)
Time of sample feeding Average processing speed	19 min 0.288 kg/min	27 min 0.22 kg/min
Start temperature of the process:	1410 °C	1419 °C
End temperature of the process:	1445 °C	1468 °C

**Table 3:** Elemental composition of vitrified samples carried out by XRF standardless analysis, values calculated to oxide form (the results have not been normalized to 100%).

Element	SiO_2_	TiO_2_	Al_2_O_3_	Fe_2_O_3_	MnO	MgO	CaO	Na_2_O	K_2_O	P_2_O_5_	SO_3_
**Sample**	%										
**No. 1**	32.5	1.4	9.4	3.5	0.05	0.4	12.4	0.3	0.2	0.03	0.1
**No. 2**	22.0	1.9	13.7	4.9	0.07	0.6	16.2	1.0	1.2	0.1	0.1

**Table 4:** Results on the leaching values of heavy metals from vitrified samples.

Element	Leaching Sample No. 2 (mg/kg)	Leaching Sample No. 1 (mg/kg)	Permissible Limiting Value for Leaching (mg/kg)
As	<0.02	<0.02	0.5
Sb	0.02	0.01	0.06
Ba	0.02	0.03	0.5
Cd	<0.01	<0.01	0.04
Cr total	0.03	<0.01	0.5
Cu	0.1	0.1	2
Ni	<0.02	0.21	0.4
Pb	<0.05	<0.05	0.5
Zn	<1	<1	4
Se	<0.02	<0.02	0.1
Mo	<0.01	0.06	0.5
Hg	<0.003	<0.003	0.01

**Table 5:** Results of the alkaline reactivity of vitrified samples.

Analyzed Sample	Alkaline Reactivity (%)	Fulfilling Requirements
No. 1	0.04 –> level 0	level 0
No. 2	0.08 –> level 0	
